# Perspectives on the semantics/pragmatics debate: insights from aphasia research

**DOI:** 10.3389/fpsyg.2023.1250170

**Published:** 2023-09-29

**Authors:** Roberto Graci, Alessandro Capone

**Affiliations:** Department of Cognitive Science, Psychology, Education and Cultural Studies (COSPECS), University of Messina, Messina, Italy

**Keywords:** semantics/pragmatics debate, aphasia, sociocultural pragmatics, communication, pragmatic enrichments

## Abstract

In the philosophy of language, there are many ongoing controversies that stem from relying too heavily on an utterance-based framework. The traditional approach of rigidly partitioning the utterance’s meaning into what is grammatically determined from what is not may not fully capture the complexity of human language in real-world communicative contexts. To address this issue, we suggest shifting focus toward a broader analysis level encompassing conversations and discourses. From this broader perspective, it is possible to obtain a more integrated view of how linguistic and extra-linguistic aspects dynamically interact and thus reconsider semantics/pragmatics dichotomy as complementary dimensions. Meaning is not confined to linguistic structures alone but emerges from the dynamic interplay of words, sociocultural knowledge, discursive situations, and psychological dispositions of speakers. Substantiating this perspective calls for embracing an interdisciplinary approach that synthesizes research from various domains, including linguistics, cognitive psychology, and philosophy of language. This paper focuses on a particularly compelling case study: aphasia. Speeches produced by individuals with aphasia represent complex scenarios where the balance between linguistic and extra-linguistic aspects is notably compromised, often to the former’s detriment. Aphasics’ productions represent a vivid example of how the interpretation of speeches can be far from involving fixed and static operations. Instead, it entails continuously reallocating cognitive resources toward the most readily available and accessible sources for the speakers. This case study ultimately demonstrates that the influence of semantic and pragmatic processes in shaping and conveying meanings displays remarkable adaptability, continuously adjusting to the ever changing demands placed upon speakers.

## 1. Rethinking the semantics/pragmatics divide

Since Morris (1972) proposed to distinguish semantics (which refers to the study of the connection between signs and meaning) from pragmatics (associated with the relationship between signs and interpreters), these terms have gained significant prominence in both philosophical and linguistic disciplines. While scholars generally agree on the distinct research objectives pursued by semantics and pragmatics, further exploration reveals many divisions and disagreements concerning the relationship between these two dimensions. Indeed, some approaches tend to lean toward a semantic understanding of language, while others emphasize pragmatic explanations. Typically, the formers view language as an abstract representation mediated by the semantics of the uttered sentences, while the others view language as a collaborative and socially embedded action, inseparable from the physical and sociocultural context. At present, these two trends have been labeled with the names “minimalism” and “contextualism” (Recanati, 2004, 2005; Bianchi, 2009).

One of the most popular ideas within minimalism is that semantics should not accommodate a large number of contextual effects or, to put it another way, semantics should impose limitations on the context dependency of the linguistic system. The method of deriving the utterance meaning follows a bottom-up direction, starting with the properties of syntactic constituents and the rules for combining them compositionally. By employing operations guided by linguistic material, it becomes possible to obtain “minimal” contextual information that forms a complete proposition. According to this perspective, pragmatics only intervenes to fill the slots left empty by semantics. The process of filling empty slots involves stable parameters relating to time, place, speaker, or domain variables. Similar views have also been identified as “indexicalism” (Stanley, 2000, 2002; Stanley and Szabó, 2000 King and Stanley, 2005).

Another prominent minimalist position is the Insensitive Semantics of Cappelen and Lepore (2005), according to which if we disambiguate ambiguous expressions and if we fix the referents of the indexical components of a sentence *S* in a context *C*, we obtain the minimal semantic content of an utterance *u*. The semantic content of an utterance is a structure that remains unaffected in all contexts, regardless of the type of speech act that the speaker performs. Cappelen and Lepore acknowledge that a speaker could assert “She is happy” with an additional or different meaning beyond the minimal proposition. However, establishing this further level of meaning goes beyond the purpose of a semantic theory and takes into account pragmatic purpose. When they mention the concept of minimal semantic content, they refer to a basis or a semantics anchor (shared by all utterances of a sentence in whatever context) from which to start interpreting the speaker’s conversational contribution. Their main idea is that communication must have content that is expressible semantically.

In general, minimalist theses try to preserve an idea of semantics as systematic and compositional as possible. They aim to develop uniform rules that apply in most cases. On the other hand, minimalist theses seek to preserve the idea that the meaning of the whole is a function of the meaning of its parts and how these parts are combined. Systematicity and compositionality guarantee a certain degree of clarity in a theory of language. The limitation of external factors to syntax and semantics makes it possible to formulate rigorous hypotheses that regularly apply from case to case.

On the contrary, proponents of contextualism contend that most sentences uttered in a context are sub-propositional, in the sense that they do not express a complete (true-conditional) proposition without the intervention of *top-down pragmatic processes* (Bach, 1994, 2004; Sperber and Wilson, 1995; Carston, 2002; Recanati, 2010). These processes occur beyond the boundaries established by syntax and lexicon and steam from dynamic interaction factors, such as the speakers’ psychological background, shared knowledge, and socio-cultural assumptions.

Some of the most radical contextualists assume that several propositions conveyed by utterances do not have an identical structure, component by component, to the linguistically articulated one but contain “unarticulated constituents” (Perry, 1993: 206). For instance, Perry argues that the statement “It is raining” expresses a proposition more complex than the overtly expressed one, as it includes the specification of the “place” where the event is occurring. Similarly, Bach explores statements like “John is ready,” where the specific context of what he is ready for (e.g., to go out, to prepare for an exam, to leave) needs to be inferred through a “completion” (Bach, 1994: 125) process. Perry and Bach strongly oppose the notion of hidden indexicality or empty constituent slots, which suggests that sentences (supposed to be) semantically under-determined contain hidden or implicit variables linked to specific linguistic items.

Conversely, proponents of indexicalism, such as King and Stanley (2005), argue that the grammar of a language is the analysis level that provides all possible explanations of meaning construction. Any context-dependency must be attributable to the presence of an *articulated constituent* – be it a pronounced indexical or a hidden variable in the logical form. As Stanley states: “for each alleged example of an unarticulated constituent, there is an unpronounced pronominal element in the logical form of the sentence uttered, whose value is the alleged unarticulated constituent” (Stanley, 2000: 410). For the author, there must be a location variable traceable to the logical structure of VP in “it is raining.” Instead, “John is ready” would contain a context-variable argument required by the adjective “ready”^1^ [for a more extensive view see also Capone’s review of Capone (2019a,b): 148].

Today, contextualism and minimalism are far from reaching a unanimous agreement. They continue to provide insights into how syntactic/semantic or pragmatic operations can contribute to forming a complete and autonomous proposition. In doing this, both positions often adopt abstract and ideological tones, overlooking the psychological plausibility of their theories and the sociocultural underpinnings of communicative practices. In this context, we claim that any theory of meaning should (1) Incorporate contemporary findings from cognitive sciences on how humans assimilate and process communicative stimuli; (2) Shift toward a more substantial reliance on *corpora*, reducing the emphasis on potential utterances in hypothetical contexts.

As regards the first aspect, proponents of minimalism and contextualism may be seen as being, up to a point, on the same wavelength. They both acknowledge the importance of lexical meaning, syntactic structures (the semantic glue that combines lexical constituents), and the role of context in adding levels of meaning. Minimalists argue that all the elements within a sentence suffice for obtaining a complete (truth-conditional) proposition, whereas contextualists argue that the semantic representation of the uttered sentence should be augmented and enriched through inferences that draw upon the extralinguistic context to be truth-evaluable. Nonetheless, both approaches reinforce a static communication model that prioritizes grammatical information. It consistently begins with exploring the syntax and lexicon of the sentence and subsequently considers the discursive circumstances to determine the appropriate direction that the interpretation must take. Conceiving that the process of deriving meaning in the complexities of everyday communication follows such a linear path seems challenging. On the other hand, contemporary findings from cognitive sciences have challenged the idea that there is a clear separation of linguistic-communicative tasks within our cognitive architecture [for a more comprehensive discussion, see Graci (2023)].

Jaszczolt (2005, 2010) has criticized this method on multiple occasions. According to the author, the analysis of utterance meaning must go beyond truth conditions and extend to the level of its position in the discourse. The grammar of a sentence should not be viewed as the initial point from which the speaker’s meaning is derived incrementally but rather as one of the numerous sources that collectively contribute to the creation of a communicative act. Rather than postulating predefined levels of interpretation and thus assessing their processing load, Jaszczolt advances the idea that there is only one level of meaning (a final representation Σ) to which different pieces of information contribute.

Several authors have raised similar doubts concerning the necessity of postulating separate and disconnected bottom-up or top-down interpretative processes. Stainton (2006), for example, argues against the existence of a hierarchy between linguistic and extralinguistic information in determining what is said. He argues that any information inferred within a communicative context is integrated into a central system. This system transforms multi-modal representations into a unified conceptual format by drawing from various cognitive systems (e.g., perceptual and linguistic). Likewise, Cummings (2005) posits that imposing predefined levels of interpretation within our mental architecture is implausible. Any data supporting semantic/pragmatic inferences does not possess properties in isolation from the whole knowledge system. Instead, access to interconnected beliefs and networked information is necessary to compute what is explicitly or implicitly conveyed. In her view, language and perception are properties of the whole mind and, therefore, cannot be confined to encapsulated information systems.

By embracing Jaszczolt’s theoretical position, which posits that a single level of meaning arises from the contributions of various information sources, we can transcend the traditional minimalism/contextualism dichotomy and view it as a complementary relationship. In the complexity of everyday communication, different stimuli of linguistic and non-linguistic nature gradually converge in the central system, yielding a unified representation. Each information source ensures that the production and understanding of meaning proceed smoothly. However, when the balance between these sources is lost to the disadvantage of syntax, the extra-linguistic factors likely assume a predominant role. In this perspective, the capacity of the linguistic system to express complete thoughts varies. This variability depends upon many aspects, including the speakers’ specific needs and preferences. Their inclination, necessity, or circumstances are pivotal in determining whether certain concepts are expressed linguistically or through contextual means.

To explore the role of speakers in fostering forms of communication that prioritize either semantics or pragmatics in the production and comprehension phase, we must redirect our focus toward genuine communicative contexts and actual discourse practices of individuals. Current research lines of socio-pragmatics follow this direction. They represent a breakpoint from the linguistic-philosophical tradition that seeks to investigate speaker meaning within an utterance-based framework. Notably, Mey (2001, 2006) has significantly supported the advancement of sociocultural perspective, particularly in his critique of speech act theory. The limitation of speech act theory lies in its narrow focus on individual actions, neglecting the collaborative nature of social activities that arise within a community. Linguistic communication is not solely the domain of individuals; instead, it emerges through a collective interplay of language rules, social norms, and environmental factors relevant to the conversation. For Mey, pragmatic acts are defined by the situational context and extralinguistic factors such as gestures and intonation rather than mere linguistic “wording.” He argues that, strictly speaking, there are no discrete speech acts as independent entities, but rather, there are speech acts that take place within a given situation:

“[t]he focus is on the environment in which both speaker and hearer find their affordances, such that the entire situation is brought to bear on what can be said in the situation, as well as on what is actually being said” (Mey, 2001: 221).

Kecskes has put forth a dynamic approach to communication, known as the SCA [Socio-Cognitive Approach, see Kecskes (2010a)], which aims to overcome the limitations of both bottom-up (from grammar to the outside world) and top-down (from discursive context to utterance meaning) approaches. A comprehensive theory should encompass a bi-directional movement that integrates both internal and external aspects of language without giving absolute priority to either. In this framework, individual cognitive factors (egocentric) and societal factors (cooperative) operate in parallel, striking a balance for optimal communication. Kecskes’ hypothesis is based on two central claims. Firstly, the speaker and the listener are equal participants in the communication process. Both draw upon their most accessible and salient knowledge to produce and understand language. Therefore, a comprehensive understanding of linguistic communication necessitates a holistic interpretation of the utterance from both the speaker’s and the listener’s perspectives. Secondly, communication is a dynamic process in which individuals are not merely constrained by societal conditions but actively shape them. While Kecskes employs a dynamic approach that considers multiple factors in the analysis of meaning, he still recognizes the crucial role played by literal meaning, as shown numerous times in interactions between foreigners living, say, in the United States or between first-language users and second-language users (Kecskes, 2008). Nonetheless, linguistic information does not always dominate all production and comprehension phases; it comes to the forefront only when specific needs arise. For instance, it can assume a priority role in supporting communication between individuals from different sociocultural backgrounds with limited shared knowledge.

Similarly, scholars like Caffi (2002) have highlighted that the essential role of pragmatics should be capturing the intricate connection between language and the communicative situation. This encompasses the dynamic interplay of evolving discourse, the unfolding linguistic actions aimed at shaping the context, and the different participant roles that shape this process. The examination of participant roles can be limited to the discourse realm, or also expanded to enclose extra-linguistic aspects. Verschueren (1999) reinforced these ideas by emphasizing that utterances, when viewed in isolation, possess high indeterminacy due to the numerous contextual configurations they can potentially align with. Avoiding the fallacy of reifying or solidifying context as a static entity is crucial. Context is dynamic and fluid, continuously influenced by various factors, and should be treated as such during analysis and interpretation (Verschueren, 1999: 111–112).

Socio-pragmatic approaches emphasize the importance of including multiple elements such as prosody, gestures, cultural knowledge, personal experiences, and the physical environment in the process of meaning-making. To fully comprehend the complexity of meaning, linguistic research should shift toward studying discursive situations that occur within culturally significant contexts [what Goffman (1974) identifies as *frames*]. Contemporary research also accentuates the importance of employing multi-method approaches encompassing natural and elicited data in investigating socio-linguistic phenomena [for a more extensive discussion, see Houser and Kádár (2021)].

## 2. When pragmatics is needed? the case of aphasia

Sociocultural investigations have revealed that the production and interpretation of a statement involve a multitude of linguistic and extra-linguistic factors. These factors do not intervene sequentially but in parallel, collaboratively shaping and transmitting the speaker’s intended meaning. Furthermore, the contribution of these factors to the determination of meaning is not fixed or static. Their balance constantly evolves in the dynamic realm of everyday communication. Changes can arise from sociocultural influences, conscious choices of speakers, or situational and physical constraints that limit access to specific sources. As a result, the interpretative load is continuously redistributed, activating appropriate interpretive processes that rely on the available resources.

The fundamental principle at play is that cognitive and situational needs may prompt a reduction in the reliance on grammatical structures and, consequently, the intervention of pragmatic inferences primarily based on context. On the other hand, other situations or needs may lead speakers to rely more on the linguistic system, limiting contextual information. This premise is essential because it takes the theoretical debate onto a natural ground. Currently, there is a certain degree of exaggeration in the under-determinacy claim. Many radical contextualists argue that the semantic content of sentences is virtually never propositional and that top-down pragmatic processes are ubiquitous. In proving this point, they focus on the enunciation of short sentences in potential contexts. However, they do not consider very long and complex sentences that can make explicit what a speaker means. Nor do they consider situations where a speaker wants to convey minimal and unenriched content. If we want a fairer view of the current theoretical positions, it is essential to differentiate between various communicative situations and consider the cognitive needs of speakers. By observing tangible cases, there are situations where the language system cannot offer great support to communicators for one reason or another. Therefore, pragmatic inferences acquire a predominant role. Conversely, there may be several situations wherein the sentences enunciated are so accurate and specific that the intervention of pragmatics in determining the propositional content is minimal (although always necessary). In the latter instances, semantic inferences assume a more significant role.

To illustrate the first type of situation, consider the case of aphasia. Aphasia is an acquired language disorder. It usually occurs following a focal lesion to the left hemisphere caused by degenerative disease, stroke, or head trauma. Language impairments in aphasia never emerge in a stable and regular way. Instead, we find a multitude of linguistic deficits that vary in qualitative and quantitative terms. In order to establish a clinical framework within which to operate, many diagnostic manuals rely on the distinction between fluent and non-fluent aphasia. The fluent/non-fluent distinction is never clear-cut but corresponds to two extremes of a continuum that sees intermediate situations. In general, the fluent pole is more characterized by lexical-semantic problems: patients make mistakes in the choice of words, repeat the same expression several times, or use inappropriate referential devices. Instead, the non-fluent pole is more characterized by difficulties of a syntactic nature. These can involve the omission of articles, grammatical words, and morphological elements.

In recent years, the development of investigation techniques in clinical pragmatics has brought an unexpected view of aphasics’ communicative competence. Most studies converge on the idea that structural language problems do not undermine the level of appropriateness of communicative behaviors. Despite limited linguistic resources, individuals with aphasia can establish shared meaning with their conversational partners and achieve their communicative goals (Olness and Ulatowska, 2011). One remarkable case study that proves this point comes from Goodwin (1995). His study focuses on Rob, an aphasic patient with an extremely limited vocabulary consisting of three words: “yes,” “no,” “and.” Despite this significant constraint, Rob demonstrates an ability to recognize interactive situations and actively participate in conversations by making his contributions at the most appropriate moment. Through nuanced variations in the pronunciation of “yes” and “no” and his use of bodily movements, Rob ably guides his conversation partners toward the intended meaning. This case study aligns with other research findings that highlight the ability of many individuals with aphasia to successfully adhere to conversational turn-taking norms (Hengst, 2003; Rhys et al., 2013). Olness and Ulatowska (2017) argue that individuals with aphasia can represent factual information about people, places, and things, thereby demonstrating their possession of narrative and referential coherence. These insights challenge the idea that the ability to express a viewpoint on a state of affairs largely depends on grammatical competence.

Mutual understanding between aphasic patients and ordinary speakers can be achieved through a synergistic integration of limited linguistic resources with other contextual cues. Even when aphasic patients produce words that deviate from their intended referential meaning, communication is still possible, as illustrated by the following case study involving an Italian patient (MacWhinney et al., 2011)^2^:

(1)

Io e mia moglie eravamo tutti e due a lavorare. E non andiamo... non andavamo da nessuna parte visto che eravamo tutti e due a lavorare […] Ma quando poi siamo andati in pensione abbiamo cominciato a **fare le visite**. E diciamo che una delle prime visite era in Egitto... poi seguito abbiamo fatto tante visite in Sardegna.

*My wife and I were both at work. And we are not going... we were not going anywhere since we were both at work […] But when we retired we started **making visits**. And let us say that one of the first visit was to Egypt... then we made many visits to Sardinia.*

Here, the patient uses the expression “making visits” instead of “travelling.” This expression is too general in the communicative situation at hand. In Italy, “making visits” can refer to various contexts beyond holidays, such as dining with a friend, undergoing a medical examination, spending time with parents, visiting a deceased person at the cemetery, or inspecting a place to ensure everything is in order. Despite “making visits” and “travelling” sharing some crucial elements,^3^ the former activates a much broader concept than the latter. However, the problem of the wide range of applications of “making visits” can be solved through a pragmatic inference similar to “narrowing” discussed by some contextualists. It would allow the recipient to process further information from the context to restrict the semantic domain of the term. In the case under examination, our understanding of retirement, well-known tourist destinations, and the ongoing conversation’s topic favor the interpretation of a *holiday trip*. In this process, the involvement of knowledge from the linguistic context is also unavoidable. The reciprocal influence between the statements “We started making visits” and “And let us say that one of our first visits was to Egypt” restricts the application of “visits” and provides clues as to the intended meaning.

Consider another case where a clinician asks an Italian patient with moderate non-fluent aphasia to share a significant life event. In response, the patient tells the clinician about his graduation day:

(2)

Eravamo in dieci e stavamo aspettando il turno. Quando sono entrata al... Otto professori... e sono stata molto molto molto emozionata. Ma erano professori che... come dire? Il professore che ho avuto la tesi con lui e erano molti. Ma tutti mi hanno messo le domande. E e poi quando siamo stati fuori. Quando ho detto grazie. E così è stato. Emozionante emozionante. E poi con tre quattro colleghe siamo andate a **sbranare [il cibo]**. Che eravamo stanche ma che avevamo questa realizzazione molto molto importante.

*There were 10 of us and we were waiting for our turn. When I entered the... Eight professors... and I was very very very excited. But they were professors who... how to say? The professor I had the thesis with, and they were many. But everyone put me questions. And then when we were out. When I said thanks. And so it was. Exciting exciting. And then with three or four colleagues we went to **tear the food**. That we were tired but that we had this very very important realization.*

In its standard meaning, “tear” refers to the act of tearing something apart or into multiple fragments with great force or intensity. The emphasis is on the vigorous and thorough destruction or fragmentation of the object in question. However, when applied to human food, this verb is not suitable as it is more specific to the actions of certain animals tearing prey apart using teeth or claws. In order for the term to align with the patient’s communicative intentions, a pragmatic inference is required. A process in line with what contextualists call loosening or broadening [see Carston (1997) for a more extensive view of this process] leads the hearer to ignore the most frequent (and therefore rigidly fixed) interpretation and extend its scope. In this case, the term “tear” undergoes a shift in meaning. It loses its specific connotation of angrily tearing something or someone apart and adopts a more generic sense of eagerly consuming or eating.

There are also instances where an aphasic patient might leave a sentence incomplete or randomly omit parts, resulting in “gaps” at the propositional level. This phenomenon is exemplified by the following case, wherein a woman with moderate non-fluent aphasia recounts her graduation ceremony experience to the clinician. When asked to describe this highly emotional event, the patient says:

(3)

Ti posso parlare della... del giorno della laurea che... sì è stato un momento **che mi ha reso...** soprattutto nel finale.

*I can tell you about... about the graduation day that... yes it was a moment **that made me...** especially in the end.*

Understanding the patient’s communicative intentions is not a difficult task despite the omission of linguistic elements. We are aware of the challenges involved in obtaining a degree and the subsequent opportunities it opens in society. This awareness stems from our common and shared experience of the world. Consequently, the joyfulness associated with a graduation ceremony holds a public, intersubjective value. Drawing upon this knowledge, we can develop a tentative interpretation of the missing part. We can imagine that the moment of graduation made the woman particularly *happy* or *excited*, especially toward the end of the ceremony when the tension subsides and the proclamation occurs.

Here is another dialog between a clinician and a man with moderate non-fluent aphasia. The patient is asked to recount a significant life event, and he chooses to narrate the period during which he had a stroke that subsequently resulted in his aphasia. His account is somewhat fragmented, but it reveals several crucial details:

(4)

Ci sono quattro cinque cose no? Che sono... lavoro. Troppo lavoro. Ho lavorato troppo. Poi mi hanno sba... hanno cambiato le medicine. E mi hanno fatto lavorare dalla sera fino alla notte. Ho bevuto un po’ di caffè un po’ di birra e ho fatto qualche giro in bicicletta. E ho bevuto un po’ di vino che mi hanno dato. Ho mangiato altre cose... quindi ho mangiato anche troppo. E ho fatto troppe cose con i miei bambini... e quindi è stata una settimana terribile... troppe cose. E quindi **mi è andato il... in tilt**.

*There are four or five things right? Which are... Work. Too much work. I worked too much. Then they got me... [they] changed my meds. And they made me work from evening until night. I drank some coffee and some beer and went on a few bike rides. And I drank some of the wine they gave me. I ate other things... then I ate too much. And I’ve done too many things with my kids… and so it’s been a terrible week… too many things. **And then my... went haywire**.*

Considering the subject matter of their discussion, we can tentatively interpret the final part of the patient’s speech by assuming that his *brain* went haywire. This interpretation draws upon our general awareness of factors that can negatively impact a person’s mental health, including excessive work, an unhealthy diet, elevated stress levels, and alcohol consumption. In this context, “my brain went haywire” aptly describes the patient’s confusion during the discussed period. It also makes sense to the combination of factors disclosed in his narrative.

Due to the challenges in constructing grammatical structures, individuals with aphasia often employ an “economy principle,” whereby they eliminate grammatical words and utilize a more simplified and straightforward emergency language (Graci, 2023). On the recipient’s side, the reduced emergency language creates the cognitive need to make sense of the communication and thus integrate, complete, or modulate what is seriously compromised through reasonings from the best explanation. In such situations, it becomes evident that the generation and comprehension of thoughts demand an elevated allocation of cognitive resources toward extra-linguistic information sources. These sources encompass personal and word knowledge, prior experiences, and sociocultural assumptions.

### 2.1. Aphasia, sub-sentential speeches and free enrichments

Aphasia is just one example of a real-world discursive context characterized by reduced linguistic resources. However, there are other situations where speakers utilize a streamlined grammatical system. For instance, the omission of function words like prepositions and articles is a hallmark of speeches produced by second language learners. Isolated terms or fragmented speech are standard in telegraphic or emergency messages. Linguistic material is often omitted in discursive practices involving “loose talks.” These involve expressions employed by ordinary speakers across numerous everyday contexts, such as:

(5)

- Point well taken.
- From Spain.
- Play pianoforte.
- Very sick.
- Battery dead.
- Nice dress.

Recently, cases like (5a–f) have captured the interest of linguists and language philosophers. The motivation behind this attention lies in the potential these expressions hold to offer proof of the existence of unarticulated constituents, thereby lending support to the contextualist perspective. Given that these expressions feature minimal linguistic input, their comprehension within a discursive context must rely heavily on pragmatic interpretative processes. These processes operate *freely* on what is said, enriching the propositional level of the utterance.

Traditionally, in linguistic literature, utterances smaller than a complete sentence have been called “fragments” (Quirk et al., 1972; Sweet, 1996). More recently, Barton and Progovac have proposed the label “nonsententials” (Barton and Progovac, 2005). Progovac believes that the difference between sentences and non-sentences should be found in the specification or (the sub-specification) notion. Nonsententials tend to have under-specified or default forms concerning Tense and Case, making the projection of a Tense Phrase superfluous. The author considers the level of the Tense Phrase as a breaking point between what can be considered a full sentence and what is not (obviously in the languages that grammaticalize Tense).

Stainton (1995, 2005) was among the first authors to introduce the discussion of fragmented speeches within the semantics/pragmatics debate. He has argued that it is possible to perform fully-fledged speech acts by employing an NP, PP, or VP. Therefore, if a boy holds up a letter and says (5b), he essentially asserts that the letter is from Spain. Stainton claims that there is no process triggered by semantics or any phonologically or syntactically suppressed material. This is just a bare expression an individual uses to convey a complete thought. Pragmatics plays a decisive role in bridging the gap between the sub-sentential speech and the speaker’s intended proposition. In his perspective, the expression in (5b) carries a definite semantic meaning (type) that remains consistent across all contexts. Nonetheless, its use in a particular communicative situation encourages the development of an additional, non-linguistically specified meaning. The same principle applies to all other instances in (5):

“Carston (1988), Recanati (1989), Searle (1978, 1980), Sperber and Wilson (1986), and Travis (1985, 1996, 1997) have all argued, contra the (purportedly) Gricean line, that there are pragmatic determinants of what is literally stated, asserted, or said which cannot be traced to elements of linguistic structure. This has become known as the thesis of pragmatic determinants of what is said. (This thesis is closely related to the idea that there exists what Perry (1986): 138 calls ‘unarticulated constituents’: a constituent of the propositional content of the speech act for which there is no corresponding constituent in the expression uttered.) Now, as first noted in Stainton (1997), sub-sentential speech provides another example of pragmatic determinants of what is stated, asserted, or claimed – at least if the pragmatics-oriented approach sketched above is the right one” (Stainton, 2005: 391–392).

As expected, the thesis on the pragmatic determination of fragmented utterances has aroused some perplexity among scholars adhering to minimalism (among the most prominent are Stanley, 2000; Ludlow, 2005). They argue that a genuine speech act must consist of a complete sentence and that there are no such things as non-sentential speeches. Any purported instance of sub-sentential speech is, in fact, a structurally self-sufficient linguistic form, which is isomorphic with the expressed proposition. According to this perspective, everything left unspoken is retrieved solely through grammatical operations, without any intervention of pragmatics. Some linguistic material remains active at the grammatical level, albeit phonologically suppressed. To address this phenomenon, minimalists have resorted to the notion of *ellipsis*.

In linguistics, it is a truism to say that sounds do not correspond directly to meanings. Instead, there is an intermediate level represented by the syntax. When a sound is emitted, it corresponds to a syntactic structure, which, in turn, corresponds to semantic content (according to the following scheme: Sound Pattern → Syntactic Structure → Semantic Content). In light of this, we can explore two paths to establish how a sound can end up abbreviating a linguistically encoded message: (a) the irregularity comes from the sound/syntax relation; (b) it comes from the syntax/semantic relation. For minimalism, the second hypothesis is unsustainable. It tends to preserve interface uniformity as much as possible - on the other hand, even the term *minimalism* was coined to suggest a “minimum” distance between syntax and semantics. Semantic compositionality must reflect syntactic combinatoriality because semantics is a purely interpretative component, and the representations it receives from the computational system (the only generative engine of language) must be necessary and sufficient to draw the correct interpretation.

Since the second path poses problems for minimalism, it is more appropriate to say that the irregularity comes from the relation between sound/syntax. Thus, when a speaker produces a sound like:

(6) /John wants a dog, but Jane does not/, it is an abbreviation of the corresponding syntactic structure.

(6a) [_S1_ [_S2_ John [_I′_ [_VP_ wants [_NP_ a dog]]]] but [_S3_ Jane [_I′_ does not [_VP_ want [a dog]]]]].

However, this technical account of ellipsis proves inadequate when applied to the instances (5a–f). Several scholars, such as Barton (1990, 2006), Stainton (2006), and Hall (2009), have effectively highlighted this point. It is worth examining their criticisms, as they are relevant to the ongoing discussion surrounding aphasia. Below, we summarize the three most robust criticisms, which, when taken together, suggest that the interpretation of gapping and fragmented sentences places a more significant interpretive burden on extra-linguistic sources.

The first substantial critique revolves around the requirement of *grammatical autonomy* that ellipsis must exhibit. The primary function of the ellipsis is to limit lexical redundancy within complex sentences and avoid repetitions. It performs an essential cohesive function, similar to replacement or anaphora. However, unlike the replacement, where we find a “placeholder” for the previous sentence reference, in the ellipsis, there is a structural hole that must be filled. That is, the elliptical sentence structure is such as to presupposes a previous or subsequent item, which serves as an indicator of the missing piece of information. Consider the following examples:

(7) /Anna brought drinks, Caterina sweets, and Valentina ice creams/.

(8) /John loves and Peter hates his mother/.

Case (7) comprises three clauses, two lacking a sound realization of the verb. The missing item is recovered starting from the predicate expressed in the first clause (to bring). This type of ellipsis is called anaphoric. In (8), however, the missing item in the first clause is made available only in the subsequent co-text: the first occurrence of the NP “his mother” remains unexpressed. This type of ellipsis is called cataphoric. The difference between (7) and (8) leads to the idea that the ellipsis involves a coordination relationship between two (or more) sentences. This relationship includes a trigger sentence, a target sentence, and an operation that unites them (Stainton, 2005). The trigger sentence is the one pronounced and available to the hearer. The second is the one that is not fully pronounced and to which the recovery operation is applied based on the trigger sentence. What can vary is the position of the two sentences and, consequently, the type of recovery operation.

The criterion of grammatical autonomy does not fit with sub-sentential speeches posited by Stainton. Consider a scenario where John spots a stunning girl at a party. He walks up to her to introduce himself and says (5f). By using the expression “Nice dress,” John asserts something like “This is a nice dress” or “Your dress is nice.” Notably, there is no preceding or subsequent linguistic material or sentences accompanying (5f) that could trigger the retrieval of the phonologically omitted content. A distinctive feature of non-sentential speeches is that they can occur *in isolation* without generating discursive anomalies. The same does not apply to elliptical structures, where the criterion of grammatical autonomy demands their coordination with a trigger sentence. While it is not possible to say “* Jane does not” at the beginning of the speech, there is nothing wrong with starting a conversation by saying “Nice dress.”

Applying the criterion of grammatical autonomy to aphasics’ productions becomes quite problematic. Individuals with aphasia face significant challenges in assembling words into phrases and sentences, including correctly applying grammatical suffixes to word stems. Consequently, their speech often deviates from the expected grammatical norms, leading to frequent discursive irregularities. Nevertheless, despite their difficulty adhering to linguistic conventions, we can still understand their speeches (or at least grasp the general meaning of their narratives, as emerged from section 2). This observation underscores that communication transcends mere grammatical competence. We can interpret the communicative intentions of an aphasic patient because our cognitive structures heavily rely on information sources that extend beyond the boundaries of “grammar.” In this sense, grammatical autonomy and correctness of uttered sentences alone are neither necessary nor sufficient conditions for effectively conveying propositional content (what is said). On several occasions, pragmatic processes assume a role far from being secondary or optional.

The second criticism revolves around the criterion of *determinacy* that elliptical structures are expected to fulfill. The recovery of the missing material in the elliptical structure is not a question of raising hypotheses or guessing, but it is an algorithmic process that, based on a linguistic input, uniquely returns a single output. In other words, the ellipsis has nothing to do with the generic implied or implicit, nor any element whose value must be derived from the context. This phenomenon occurs precisely because the explicit (trigger) sentence furnishes the necessary content to fill in the missing fragment. However, we cannot uniquely identify the omitted elements when dealing with sub-sentential speeches such as (5a–f). For instance, candidates for the unpronounced elements in (5b) might be “*This is* from Spain,” “*The letter I am holding is* from Spain,” or “*This letter is* from Spain.” The characteristic of sub-sentential speeches to prompt the development of indeterminate (albeit propositional) contents aligns seamlessly with the contextualism perspective on free enrichment. Indeed, many instances of explicatures, implicatures, or unarticulated constituents exhibit a degree of flexibility when it comes to aligning the speaker’s thoughts with the inferences drawn by the listener. Demanding a strict duplication of thoughts is unrealistic and unnecessary for a theory of meaning. A one-to-one correspondence between the proposition intentionally conveyed by the speaker and that the listener infers is not even a prerequisite for effective communication. What matters is that the two propositions are similar enough for communication to proceed in the best way.

At a higher level of analysis, the idea that all constituents of the inferred proposition must be traceable to the logical form of the uttered sentence is untenable. The determination of the sentence’s logical form lacks psychological reality. While it may be appealing for a semantic approach striving for systematicity and predictability, it remains an essentially retrospective exercise. This observation becomes even more evident when considering the speeches produced by individuals with aphasia who exhibit significant linguistic structure deficits. Individuals with aphasia produce fully-fledged non-sentential speeches, from which the propositional truth-conditional content emerges through pragmatic enrichments. These enrichments are not constrained by linguistic forms but arise independently and are influenced by contextual factors. Their motivation lies in safeguarding the interlocutor’s rationality and making sense of his/her production. Within this framework, debating whether the graduation ceremony made the girl “happy” or “excited” in example (3) becomes a trivial matter. We can intuitively discern that she experienced a profound and positive emotion, and this understanding is sufficient for us to grasp the essence of her narrative.

As Hall (2009) notes, the criterion of determinacy (although demanded by the elliptical structure) fails to establish a clear demarcation between semantics and pragmatics. In a broader sense, indeterminacy appears to be an inherent and pervasive feature of human language. This indeterminacy is not confined solely to pragmatic aspects but also permeates linguistically driven processes - consider the quantifier “some.” Its use in a real communicative context rarely provides a precise or unambiguous indication of the *quantity* intended by the speaker.

The final criticism stems from cognitive sciences and involves constraining the process of retrieving missing content within a specialized language module. According to this perspective, when someone hears an elliptical structure, s/he immediately recovers the phonologically suppressed part without drawing upon extra-linguistic resources. We have previously addressed the inherent challenge of isolating various sources of information into self-sufficient and autonomous systems. For example, one would have no way of knowing which NP the speaker has in mind without observing what the utterer of “from Spain” holds. It is difficult to argue that the listener can retrieve parts of the unexpressed propositional content without relying on the entire visual scene, word knowledge, and other sensory stimuli. For Stainton, in this case, the central system receives a concept related to the displayed letter sourced from the visual module (i.e., the argument). This concept is integrated with the other inputs stemming from the linguistic system, giving rise to a representation in a “language of thought” (Stainton, 2006: 156–157). When considering aphasic speech, presumably grasping communicative intentions involves a broader spectrum of extra-linguistic sources, including personal knowledge, sociocultural assumptions, and shared background information.

## 3. A twofold complementarity

Broadening the horizon of investigation reveals a mutually supportive relationship between semantics and pragmatics. When one information source is lacking, the interpretative load is dynamically redistributed to the remaining ones, fostering interconnected inferences.

Admittedly, the cases where utterances based on sentential meaning are self-sufficient are relatively rare. They could encompass expressions of analytic truths based on *a priori* knowledge (e.g., “whales are mammals; lions are dangerous animals”). However, pragmatic intrusions seem unavoidable even in the most straightforward instances of language interpretation encompassing synthetic propositions. Consider the statement, “The lion attacked me.” To utter this sentence, the speaker must have survived the attack, implying that this particular lion was not so dangerous. Furthermore, we can infer that the speaker is selecting a specific lion from a group and that there was an isolated event in which the lion attacked the individual. All these nuanced details are crucial for a comprehensive understanding of the described situation.

The most intriguing scenarios emerge when we delve into legal language. Lawmakers must ensure clarity, avoid ambiguity, and prevent incoherent interpretations. Rationality dictates that we interpret the law in ways that maintain coherence, align with the law’s underlying purpose, and exhibit consistency. Despite meticulous attention to form and content, lawmakers often articulate provisions demanding careful internal evaluation. For example, using pronominals is an indispensable facet of legal writing. Consider the statement, “The law punishes individuals who, when prosecuted, eliminate evidence of their guilt.” In this context, “their” must be an anaphoric reference for “individuals” in the first clause for the law to function without contradictions. Treating it as an exophoric pronominal would imply a scenario where everyone erases evidence of someone else’s guilt, resulting in a different offense from what the law originally intended. This underscores the essential role of pragmatics in interpretation and reaffirms that there are no foolproof methods to draft a law that entirely eliminates the need for pragmatic interpretation.

If we wanted to explore another scenario where the focus is more on linguistic input, we could consider scientific texts. These texts are intended for an indefinite audience and are predominantly disseminated through articles and books. Unlike face-to-face conversations, the medium through which scientific results are conveyed does not provide access to physical, environmental, and extra-linguistic cues. Consequently, the language system becomes the primary source for transmitting information. Moreover, scientific texts (such as legal texts) require accuracy, objectivity, clarity, specificity, and reliance on reliable sources. When reporting a state of affairs in these situations, speakers often employ repetitive use of terms and include apposition clauses to specify and describe the discursive context as comprehensively as possible. They strive to avoid ambiguity and undesirable interpretations through extensive use of syntax and lexicon (although even in this case, pragmatic intrusions are inevitable).

Semantics and pragmatics are like the front and back of a sheet of paper: one cannot cut out a piece without cutting out both sides. Likewise, it is impossible to entirely isolate the semantic interpretation from the pragmatic one, or vice versa, in any discourse. What changes are the situations where the interpretive burden weighs more heavily on the linguistic system (mainly activating syntactic/semantic processes) and those where the burden weighs more on extra-linguistic sources (necessitating a pragmatic analysis). This dynamic interplay between semantics and pragmatics can be described as synchronic complementarity since it applies to actual discursive contexts.

Another form of complementarity exists between the semantic and pragmatic dimensions, which manifests through temporal changes. This interplay can be characterized as diachronic complementarity, reflecting the evolving nature of language and communication over time. The short linguistic forms we examined in paragraphs 2 and 2.1 are different in nature and certainly dictated by distinct needs and (sociocultural, physical, and situational) constraints. While expressions such as “Battery dead” or “Nice dress” are more familiar to us and give rise to an immediately accessible interpretation, the fragmented speeches of aphasic patients are less recurrent and require longer processing times. Some short linguistic forms can be perpetuated in a linguistic community to such an extent that they grammatically encode even propositional content that is not explicitly expressed. Consider the case of conversational routines (CRs; Columas, 1981), such as “no problem,” or situational-bound utterances (SBUs; Kecskes, 2010b), such as “I’m just looking” in response to a salesperson. Both types of expressions can be replaced by free counterparts such as “I see no problem in satisfying your request,” or “I’m just looking at the merchandise.” However, CRs and SBUs allow speakers to save mental energy: although their general sense is compatible with more extended linguistic forms, their literal content is less. It happens because, in CRs and SBUs, much of the unexpressed propositional content is grammatically encoded and fixed through usage conventions. A similar principle applies to numerous pragmatic inferences discussed in the literature, such as “I had breakfast,” which defaults to “I had breakfast *today*.” These observations prompted Devitt (2018) to argue that various instances of sub-sentential speeches, such as (5a–f), rely on semantic conventions.

While it is true that some short linguistic forms may settle in a community and acquire conventional status, we must not lose sight of the origin of this process and its purely pragmatic reason. As Capone (2010) argued in his treatment of explicatures, there is a productive circle between semantics and pragmatics: pragmatic expansions intruding into the linguistic system can become exceptionally advantageous for speakers (it is not enough that these linguistic forms are recurring). The more a conceptual enhancement assumes importance for a community, the more it goes through a grammaticalization process and mimics semantic resources. On the other hand, the linguistic system needs pragmatic expansion to transcend the possibilities given by semantics and open up to new creative ways of communicating thoughts. Aphasia exemplifies the creative process related to the pragmatic expansion of the linguistic system since there is no systematic procedure or regularity of use, such that it is possible to make universal predictions (across different contexts) on the generation and recovery of the missing content. In this situation, the omission of linguistic material has a certain degree of unpredictability and does not tie to any discursive praxis or semantic convention. Despite this, sources of information external to language can play a predominant role in guiding the hearers toward reconstructing thoughts these patients want to convey in a given situation.

## 4. Conclusion

Nowadays, the semantics/pragmatics debate revolves around abstract issues. One of the most relevant is whether or not the grammar of a language provides all the constituents that carry meaning. In giving an answer, both minimalists and contextualists start their analyses with a potential utterance of a sentence. Then, they analyze its semantic representation to determine if this is sufficient to express a complete thought. Such an approach suffers from some limitations to the extent that it excludes the human factor. When we delve into the role of the human factor in utterance interpretation, we underscore the necessity for pragmatics to encompass multifaceted knowledge. This entails that when we analyze the speeches of individuals with conditions such as aphasia, we must delve deep into and gain a profound understanding of all pertinent sources of knowledge that contribute to how they communicate in their unique manner. To attain a comprehensive perspective, we should explore multiple facets of the individual’s life, including their personal history, the nature of their disease, family background, and habitual communication patterns. Only through such an encompassing approach can we discern that, in some situations, constraints stemming from the physical and situational factors shift the interpretative burden toward the linguistic system, predominantly activating syntactic and semantic interpretative processes. Conversely, in other scenarios, a greater burden falls on extra-linguistic sources, prompting pragmatic interpretative processes to come to the forefront.

By integrating real discursive situations in into the analysis of meaning, many theoretical issues revolving around the semantics/pragmatics debate become less relevant. One of them is whether grammar is the level of analysis that contains all the clues to derive a complete proposition. Even assuming this is the case, this does not undermine the idea that representations from the linguistic system alone are insufficient to convey helpful content from a communicative point of view. After all, it does not matter much if the logical form of the uttered sentences returns a minimal (but complete) proposition such as “it is raining in a place x” or “John is ready to x.” In any case, some sort of enrichment or adjustment of the conceptual representation provided by semantics is necessary to realize what a speaker intends to communicate in an actual context. In this light, attempting to separate language from the individuals who generate it and analyzing it as an autonomous and independent entity proves unproductive. Our language’s organization invariably evokes complex needs and demands, which can only be understood by shifting to a higher-level representation. At this higher level, linguistic sources are integrated with sensory information and sociocultural background assumptions.

## Data availability statement

The original contributions presented in the study are included in the article/supplementary material, further inquiries can be directed to the corresponding author.

## Ethics statement

Written informed consent was obtained from the individual(s) for the publication of any potentially identifiable images or data included in this article.

## Author contributions

The article is the result of a collaborative study and shared reflection. Paragraphs 2 and 3 were written by RG. Paragraphs 4 and 5 were written by AC. All authors wrote paragraph 1. All authors contributed to the article and approved the submitted version.

## Footnotes

^1^However, the matter is not straightforward since even unarticulated constituents may involve syntax elements, incorporating a comprehensive understanding of empty categories and implicit arguments. Consider an example from Roeper (1987:268–269): “The ship was sunk to collect the insurance.” This sentence can be analysed in the following way: The ship was intentionally sunk by the individuals who carried out the sinking (referred to as the agents of the sinking), with the purpose of enabling the agents of the sinking to collect the insurance. Syntacticians like Roeper (1987) propose the existence of an implicit argument (I.A.) in such sentence structures. In this specific example, the implicit argument represents the agents of the sinking. Although not explicitly mentioned in the sentence, their existence is implied by the deliberate action of sinking the ship. Recognizing the presence of this implicit argument assists in comprehending the intended meaning of the uttered sentence and sheds light on the underlying motivations behind the action.

^2^The examples provided in this part come from AphasiaBank^®^, a database created to promote research on aphasia. I thank Davida Fromm and Brian Macwhinney for providing me with valuable information on the database.

^3^A person who “makes visits” moves from his/her usual residence and then returns. Like a holiday trip, this move is temporary and ends relatively quickly.
